# A novel prognostic model for patients with colon adenocarcinoma

**DOI:** 10.3389/fendo.2023.1133554

**Published:** 2023-02-27

**Authors:** Chengliang Yin, Wanling Wang, Wenzhe Cao, Yuanyuan Chen, Xiaochun Sun, Kunlun He

**Affiliations:** ^1^ Medical Big Data Research Center, Medical Innovation Research Division of Chinese PLA General Hospital, Beijing, China; ^2^ National Engineering Research Center for Medical Big Data Application Technology, Chinese People's Liberation Army (PLA) General Hospital, Beijing, China; ^3^ Institute of Geriatrics, The Second Medical Center & National Clinical Research Center for Geriatrics Diseases, Beijing Key Laboratory of Research on Aging and Related Diseases, State Key Laboratory of Kidney Disease, Chinese People's Liberation Army (PLA) General Hospital, Beijing, China; ^4^ Medical School of Chinese People's Liberation Army (PLA), Beijing, China; ^5^ Key Laboratory of Biomedical Engineering and Translational Medicine, Ministry of Industry and Information Technology, Medical Innovation Research Division of Chinese People's Liberation Army (PLA) General Hospital, Beijing, China; ^6^ National Medical Products Administration Key Laboratory for Research and Evaluation of Artificial Intelligence Medical Devices, Chinese People's Liberation Army (PLA) General Hospital, Beijing, China

**Keywords:** colon adenocarcinoma, SEER, risk factors, prognosis, nomogram

## Abstract

**Background:**

Colon adenocarcinoma (COAD) is a highly heterogeneous disease, which makes its prognostic prediction challenging. The purpose of this study was to investigate the clinical epidemiological characteristics, prognostic factors, and survival outcomes of patients with COAD in order to establish and validate a predictive clinical model (nomogram) for these patients.

**Methods:**

Using the SEER (Surveillance, Epidemiology, and End Results) database, we identified patients diagnosed with COAD between 1983 and 2015. Disease-specific survival (DSS) and overall survival (OS) were assessed using the log-rank test and Kaplan–Meier approach. Univariate and multivariate analyses were performed using Cox regression, which identified the independent prognostic factors for OS and DSS. The nomograms constructed to predict OS were based on these independent prognostic factors. The predictive ability of the nomograms was assessed using receiver operating characteristic (ROC) curves and calibration plots, while accuracy was assessed using decision curve analysis (DCA). Clinical utility was evaluated with a clinical impact curve (CIC).

**Results:**

A total of 104,933 patients were identified to have COAD, including 31,479 women and 73,454 men. The follow-up study duration ranged from 22 to 88 months, with an average of 46 months. Multivariate Cox regression analysis revealed that age, gender, race, site_recode_ICD, grade, CS_tumor_size, CS_extension, and metastasis were independent prognostic factors. Nomograms were constructed to predict the probability of 1-, 3-, and 5-year OS and DSS. The concordance index (C-index) and calibration plots showed that the established nomograms had robust predictive ability. The clinical decision chart (from the DCA) and the clinical impact chart (from the CIC) showed good predictive accuracy and clinical utility.

**Conclusion:**

In this study, a nomogram model for predicting the individualized survival probability of patients with COAD was constructed and validated. The nomograms of patients with COAD were accurate for predicting the 1-, 3-, and 5-year DSS. This study has great significance for clinical treatments. It also provides guidance for further prospective follow-up studies.

## 1 Introduction

Colon adenocarcinoma (COAD) is an aggressive primary intestinal malignancy (1) that ranks fourth (6.1%) and fifth (5.8%) in morbidity and mortality, respectively (2). Furthermore, this disease is genetically heterogeneous. China is ranked first in the world in terms of new cancer cases and deaths (3, 4). In China, more than 380,000 new cancer cases were projected to be discovered in the colon and rectum annually (5). It could be seen that the global burden of cancer, including COAD, is rising, and cancer is on the verge of becoming the leading cause of death in the 21st century (6). Therefore, discovering new therapeutic strategies for COAD is of great significance.

Current knowledge of COAD is from small series and mostly from retrospective studies or individual case reports. Studies on COAD focusing on the survival and treatment of large populations have not been reported yet. The SEER (Surveillance, Epidemiology, and End Results) database offers favorable resources for the study of malignancies such as COAD for those limited to clinical trials or prospective data (7). This database using retrospective analysis represents the latest and largest COAD cohort in the literature.

A nomogram is used to calculate the possibility of clinical events using complex computational formulas. A nomogram is displayed graphically, with each clinical or laboratory indicator being listed separately and can be scored independently. The probability of clinical events can then be determined according to the cumulative scores of all variables (8–10). With the help of a nomogram, clinicians can assess the risks of survival, personalize treatment plans, optimize treatment strategies, and actively conduct follow-ups (11, 12).

In this study, the SEER database was used to depict the survival tendencies and the prognostic risk factors for COAD. We characterized the independent prognostic factors that were related to COAD and constructed a prognostic nomogram that could help oncologists accurately estimate prognosis and guide individualized treatments.

## 2 Materials and methods

### 2.1 Patients

The data of patients diagnosed with COAD between January 1, 2004 and December 31, 2015, were extracted from the SEER database through the SEER*Stat tool (7, 13). A total of 347,418 patients with COAD were enrolled in this study. Patients were excluded if their demographic or clinicopathological data, as well as follow-up, were incomplete. The following demographic variables and clinicopathological characteristics were included: age, gender, race, site_recode_ICD (International Classification of Diseases), grade, CS (Collaborative Stage)_tumor_size, CS_extension, and metastasis. To examine survival in COAD, we categorized patients with COAD based on age: <45, 45–59, 60–74, and ≥75 years. Site_recode_ICD is a recode based on primary site and ICD-O-3 histology, which included the large intestine, colon, appendix, cecum, and rectum. Grade consists of four categories: well differentiated, moderately differentiated, poorly differentiated, and anaplastic. CS_tumor_size is information on the tumor size, while CS_extension is information on the extension of the tumor. Metastasis is information on distant metastasis. Overall survival (OS) is defined as the time interval from diagnosis to death regardless of any cause, while disease-specific survival (DSS) is the time interval from diagnosis to death for patients with COAD. The patients weredivided into a training group and a validation group at a ratio of 7:3.

### 2.2 Univariate and multivariate Cox analyses

The incidence rates of COAD were estimated per 100,000 individuals and age-adjusted to the 2000 US Standard Population using SEER*Stat (version 8.3.2). The annual percentage changes (APCs) were calculated using the National Cancer Institute Joinpoint regression analysis scheme (version 4.5.0.1).

Univariate and multivariate analyses were performed to identify the related-risk factors. Univariate Cox analysis was used to analyze the occurrence relationship and the age, gender, race, site_recode_ICD, grade, CS_tumor_size, CS_extension, and metastasis. Using the results from the univariate analysis, multivariate analysis was conducted to validate the independent risk factors. Estimated DSS and OS were determined using Kaplan–Meier analysis and were compared using the log-rank test. Both univariate and multivariate analyses used a Cox regression model.

### 2.3 Statistical analyses

DSS was analyzed using the “forestplot” R package to present the *p*-value, hazard ratio (HR), and 95% confidence interval (95%CI) of each variable. Based on the results of the Cox regression analysis of patients with COAD, the final multivariate Cox regression model was visualized using the nomograms to predict the 1-, 3-, and 5-year DSS and OS. Harrell’s concordance index (C-index) was calculated to assess the performance of the nomogram. This index could expound the discrimination between a patient’s predicted and actual survival (14).

Both clinical prediction model calibration plots and receiver operating characteristic (ROC) curves were plotted, with the ROC curves being used to estimate the prediction performance and the validation set used for external validation (15, 16). The higher the area under the ROC curve (AUC), the better the prognostic accuracy. On the other hand, decision curve analysis (DCA) plotted the net benefit (NB), which was used to assess the clinical utility value (17, 18). Moreover, clinical impact maps were drawn to estimate the number of high-risk patients for each risk threshold (18). Calibration curves were also constructed for quantification. A nomogram was constructed in the training set. All statistical analyses were carried out using R software. The R packages mainly used in the analyses included ggplot2, survival, survminer, rms, and rmda. A *t*-test was performed to analyze the quantitative variables, while the chi-square test was used for qualitative data. A *p*-value <0.05 was considered indicative of statistical significance.

## 3 Results

### 3.1 Patient baseline characteristics

After applying the inclusion and exclusion criteria, and removing missing values, the study finally identified 104,933 patients with COAD diagnosed from 2004 to 2015. The baseline characteristics were in a ratio of 7:3 and were classified into a training group (*n* = 73,454) and a validation group (*n* = 31,479). The training and validation groups showed no statistically significant difference (*p* > 0.05). The detailed results are shown in **Table 1**. The total study population included 51,360 women and 53,573 men. The follow-up study duration ranged from 22 to 88 months, with an average of 46 months.

**Table 1:** Baseline characteristics of patients with alive and dead.

Characteristics	Total(n = 104933)	Training cohort(n = 73454)	Validation cohort(n = 31479)	P value
Age, n (%)				0.925
<45	6689(6.4)	4661(6.3)	2028(6.4)	
45-59	25853(24.6)	18081(24.6)	7772(24.7)	
60-74	36527(34.8)	25584(34.8)	10943(34.8)	
≥75	35864(34.2)	25128(34.2)	10736(34.1)	
Gender, n (%)				0.904
Female	51360(48.9)	35943(48.9)	15417(49.0)	
Male	53573(51.1)	37511(51.1)	16062(51.0)	
Race, n (%)				0.723
Black	12817(12.2)	8955(12.2)	3862(12.3)	
White	76473(72.9)	53508(72.8)	22965(73.0)	
Other	15643(14.9)	10991(15.0)	4652(14.8)	
Site_recode_ICD, n (%)				0.705
Large Intestine	1328(1.3)	942(1.3)	386(1.2)	
Colon	62788(59.8)	44018(59.9)	18770(59.6)	
Appendix	2093(2.0)	1465(2.0)	628(2.0)	
Cecum	17506(16.7)	12252(16.7)	5254(16.7)	
Rectum	21218(20.2)	14777(20.1)	6441(20.5)	
Grade, n (%)				0.885
Grade I	9261(8.8)	6490(8.8)	2771(8.8)	
Grade II	73414(70.0)	51374(69.9)	22040(70.0)	
Grade III	19839(18.9)	13912(18.9)	5927(18.8)	
Grade IV	2419(2.3)	1678(2.3)	741(2.4)	
CS_tumor_size(mm), n (%)				0.109
25-50	47053(44.8)	33066(45.0)	13987(44.4)	
>50	43923(41.9)	30705(41.8)	13218(42.0)	
<25	13957(13.3)	9683(13.2)	4274(13.6)	
CS_extension, Median (IQR)	400.0 (400.0-455.0)	400.0 (400.0-455.0)	400.0 (400.0-455.0)	0.505
Metastasis, n (%)				0.579
M0	84856(80.9)	59367(80.8)	25489(81.0)	
M1	20077(19.1)	14087(19.2)	5990(19.0)	
DSS, n (%)				1.000
No	72207(68.8)	50545(68.8)	21662(68.8)	
Yes	32726(31.2)	22909(31.2)	9817(31.2)	
Survival_months,Median (IQR)	46.0 (22.0-88.0)	46.0 (22.0-88.0)	46.0 (22.0-88.0)	0.897

IQR, interquartile range.

“Other” in the Race: American Indian (AK Native) and Asian (Pacific Islander).

### 3.2 Univariate Cox and risk factors for COAD patients

**Figure 1:**
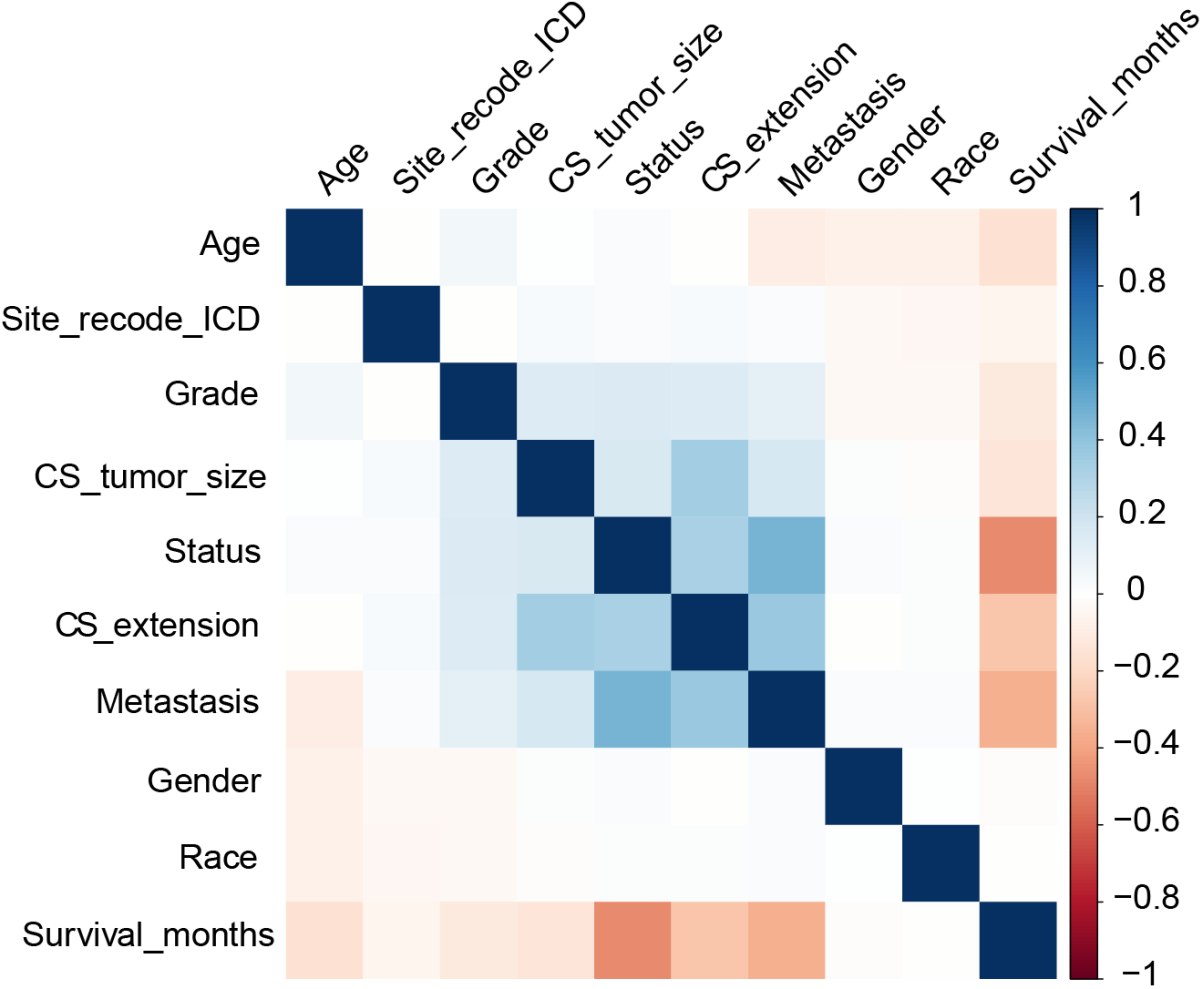
Correlations between clinical indicators in DSS.

A correlation analysis between the clinical indicators was conducted. Survival_months and status showed the most significant correlation for DSS (**Figure 1**), and metastasis and Survival_months had the most significant correlation for OS (**Supplementary Figure S1**). Univariate Cox analysis was performed to identify the related risk factors. The extracted variables in the training set showed that age, gender, race, site_recode_ICD, grade, CS_tumor_size, CS_extension, and metastasis were prognostic factors (*p* < 0.05) (**Table 2**).

**Table 2:** Univariate Cox regression results in DSS.

Univariate Cox analysis	HR	95%CI		*p*-value
		Lower	Upper	
Age (years)				
≥75	Reference			
<45	0.755	0.714	0.798	<0.001
45–59	0.728	0.703	0.753	<0.001
60–74	0.742	0.720	0.766	<0.001
Gender				
Female	Reference			
Male	1.088	1.060	1.116	<0.001
Race				
Black	Reference			
White	0.783	0.754	0.813	<0.001
Other	0.761	0.725	0.799	<0.001
Site_recode_ICD				
Appendix	Reference			
Cecum	1.409	1.264	1.570	<0.001
Colon	1.235	1.111	1.372	<0.001
Intestine	2.271	1.971	2.617	<0.001
Rectum	1.314	1.179	1.463	<0.001
Grade				
Grade I	Reference			
Grade II	1.862	1.751	1.981	<0.001
Grade III	3.326	3.117	3.549	<0.001
Grade IV	3.540	3.221	3.891	<0.001
CS_tumor_size				
<25	Reference			
>50	3.212	3.042	3.392	<0.001
25–50	2.117	2.003	2.237	<0.001
CS_extension	1.003	1.003	1.003	<0.001
Metastasis				
M0	Reference			
M1	7.506	7.307	7.711	<0.001

“Other” in race denotes American Indian (AK Natives) and Asian (Pacific Islanders).

HR, hazard ratio; CI, confidence interval

**Figure 2:**
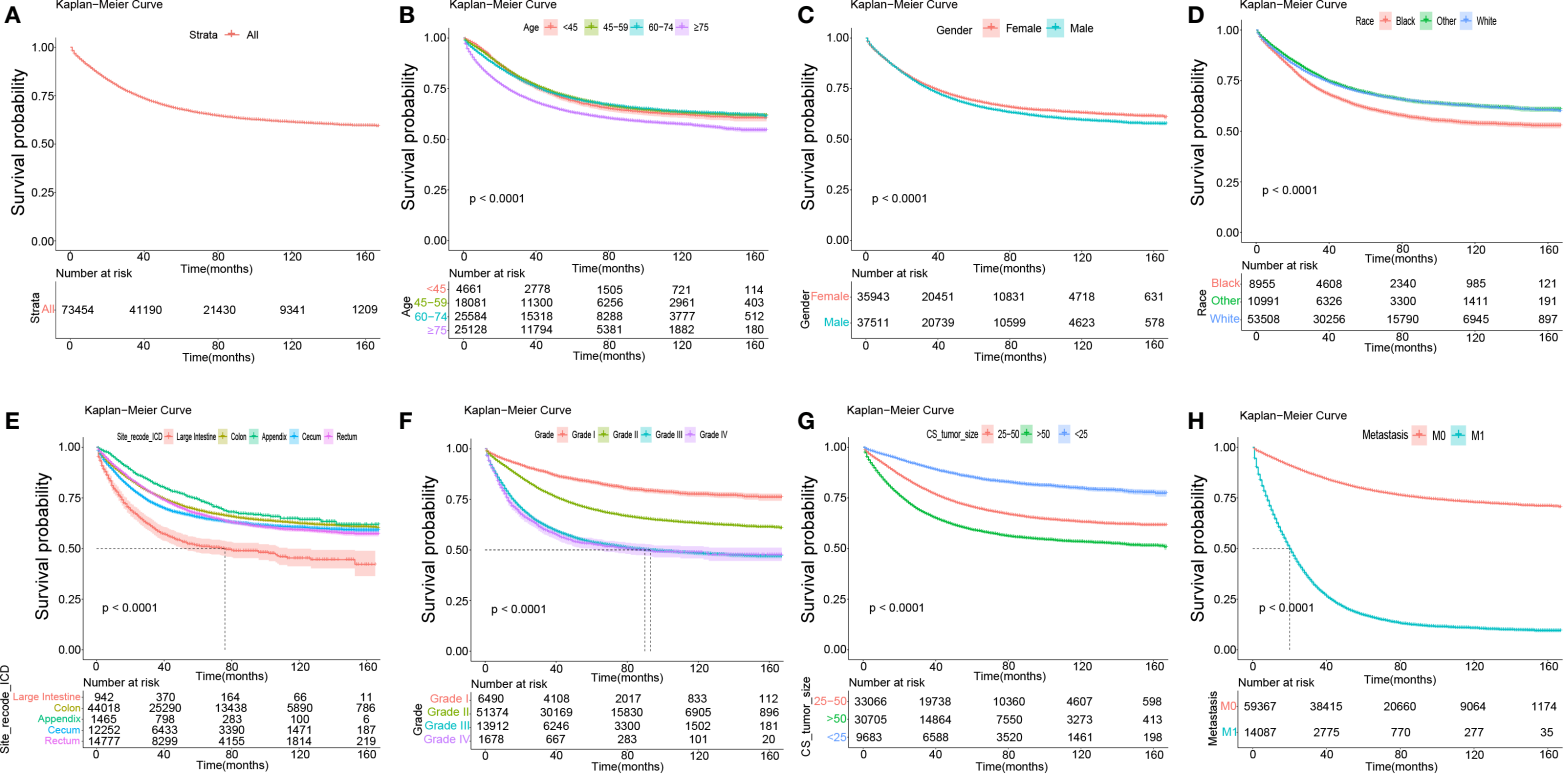
The Kaplan-Meier survival analysis in total, age, gender, race, site_recode_ICD, grade, CS_tumor_size, CS_extension and metastasis. **(A)** The survival curves of all DSS patients in the training data set. **(B)** The survival curves of age in the training data set. **(C)** The survival curves of gender (Female; Male) in the training data set. **(D)** The survival curves of race (Black; White; Other: American Indian (AK Native) and Asian (Pacific Islander), et al. ) in the training data set. **(E)** The survival curves of site_recode_ICD (Large Intestine; Colon; Appendix; Cecum; Rectum) in the training data set. **(F)** The survival curves of grade (Grade I; Grade II; Grade III; Grade IV) in the training data set. **(G)** The survival curves of CS_tumor_size (25-50; >50; <25) in the training data set. **(H)** The survival curves of metastasis (M0; M1) in the training data set.

**Figure 2A** presents the survival status of all included patients with COAD. The Kaplan–Meier survival analysis showed that those aged ≥75 years had shorter DSS compared to younger participants (**Figure 2B**). Male gender was significantly associated with shorter DSS compared to female gender (**Figure 2C**). Black patients were significantly associated with the shortest DSS compared to patients of other races (**Figure 2D**). In terms of site_recode_ICD, the large intestine was significantly associated with the shortest DSS, while the appendix was significantly associated with a higher DSS compared to the other sites (**Figure 2E**). Early stage (stages I and II) was significantly associated with higher DSS compared to other stages in site_recode_ICD (**Figure 2F**), and the risk increases with grade. With regard to site_recode_ICD, size >50 in CS_tumor_size was significantly associated with shorter DSS, while tumor size <25 was significantly associated with a higher DSS (**Figure 2G**). M1 metastasis was significantly associated with shorter DSS compared to M0 metastasis (**Figure 2H**). These results were consistent with the results for DSS in the validation cohort (**Supplementary Figure S2**). We also performed the Kaplan–Meier survival analysis for OS, which showed the same trends of the prognostic factors (i.e., age, gender, race, site_recode_ICD, grade, CS_tumor_size, CS_extension, and metastasis) (**Supplementary Figures S3**, **S4**).

### 3.3 Multivariable Cox regression and forest plot

**Figure 3:**
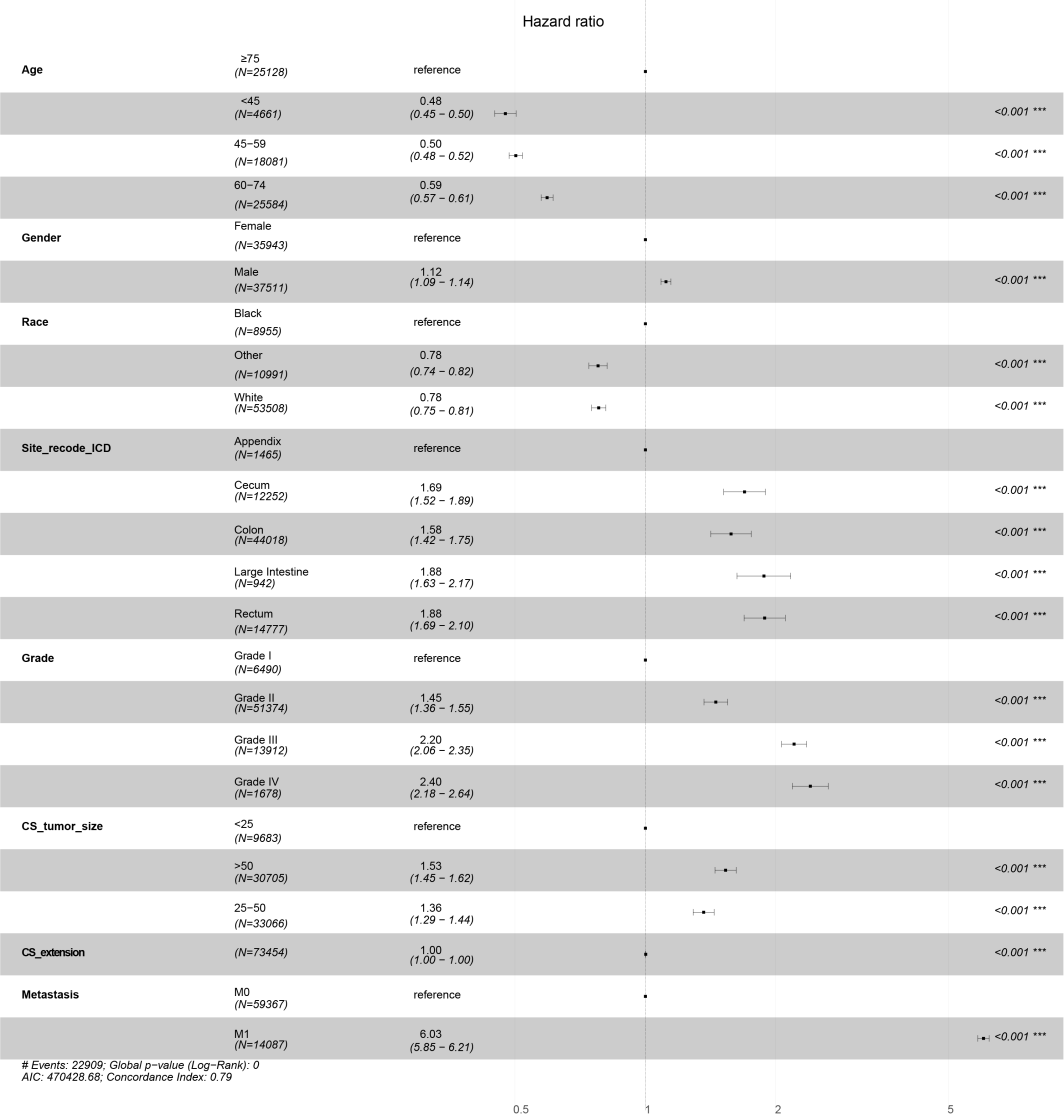
Forest plots of DSS in training data set.

Applying multivariable Cox regression on the results of the variables from the univariate analysis, eight independent prognostic factors were screened out, namely, age, gender, race, site_recode_ICD, grade, CS_tumor_size, CS_extension, and metastasis. All variables showed statistical significance for both DSS and OS (**Table 3**, **Supplementary Table S1**). The HRs of age and race were lower than those predicted for DSS (**Table 3**), which was consistent with the results of OS (**Supplementary Table S1**). On the other hand, the HRs of gender, site_recode_ICD, grade, CS_tumor_size, CS_extension, and metastasis were higher than 1 as risk factors for both DSS and OS (**Table 3**, **Supplementary Table S1**). Furthermore, forest plots were drawn using these eight independent prognostic factors, as shown in **Figure 3** for DSS and **Supplementary Figure S5** for OS. The forest plots showed that age, gender, race, site_recode_ICD, grade, CS_tumor_size, CS_extension, and metastasis were independent risk factors.

**Table 3:** Multivariable analysis of DSS in the training cohort.

Variable	HR	95%CI		*p*-value
		Lower	Upper	
Age (years)				
≥75	Reference			
<45	0.475	0.449	0.503	<0.001
45–59	0.502	0.485	0.520	<0.001
60–74	0.593	0.575	0.612	<0.001
Gender				
Female	Reference			
Male	1.115	1.086	1.145	<0.001
Race				
Black	Reference			
White	0.780	0.751	0.810	<0.001
Other	0.777	0.741	0.816	<0.001
Site_recode_ICD				
Appendix	Reference			
Cecum	1.693	1.516	1.892	<0.001
Colon	1.576	1.415	1.755	<0.001
Large Intestine	1.877	1.627	2.166	<0.001
Rectum	1.884	1.689	2.102	<0.001
Grade				
Grade I	Reference			
Grade II	1.453	1.365	1.548	<0.001
Grade III	2.203	2.063	2.354	<0.001
Grade IV	2.403	2.184	2.643	<0.001
CS_tumor_size				
<25	Reference			
>50	1.531	1.447	1.620	<0.001
25–50	1.363	1.289	1.441	<0.001
CS_extension	1.002	1.002	1.002	<0.001
Metastasis				
M0	Reference			
M1	6.028	5.849	6.214	<0.001

“Other” in race denotes American Indian (AK Natives) and Asian (Pacific Islanders).

HR, hazard ratio; CI, confidence interval

### 3.4 Nomogram construction and model validation

**Figure 4:**
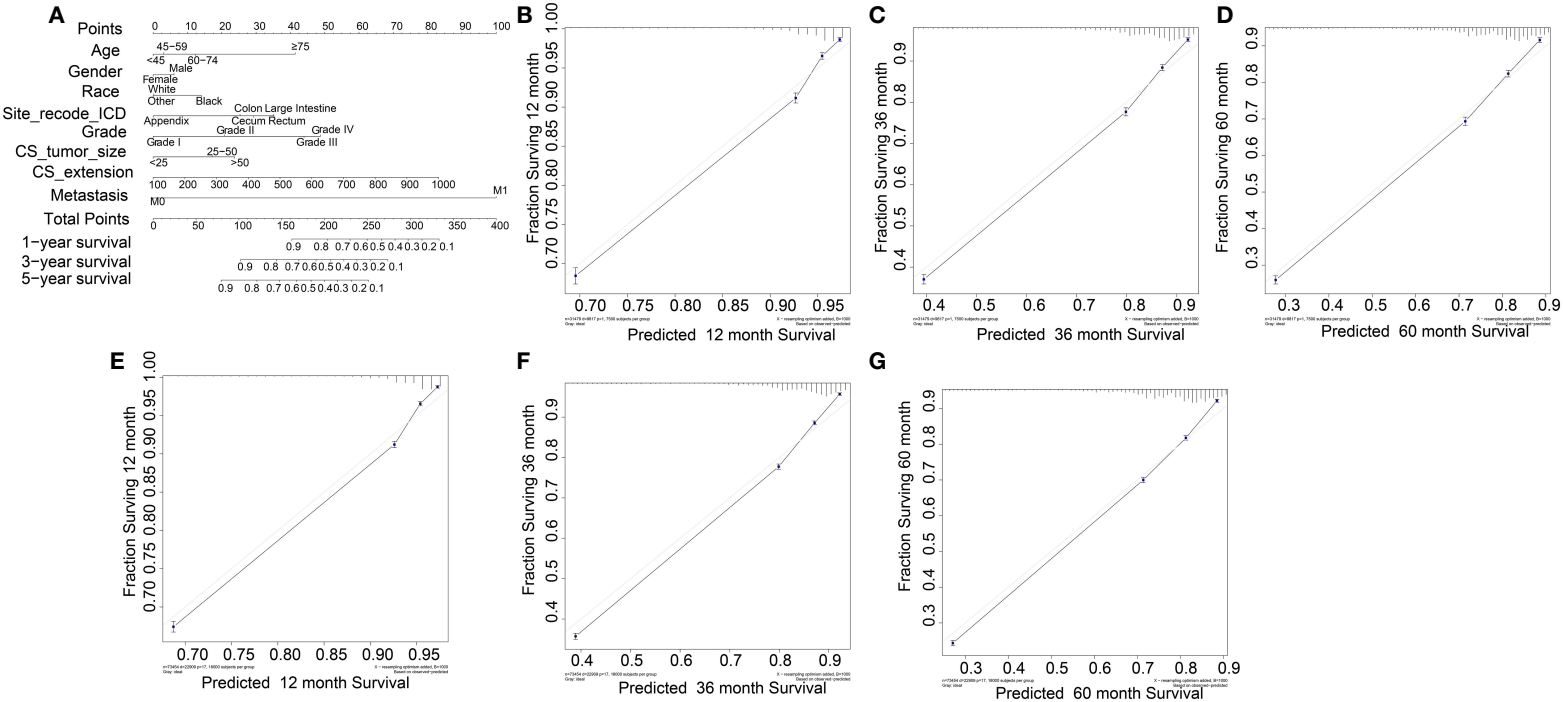
Nomogram for COAD patients. **(B-D)** and **(E-F)** were its training data sets and the validation data sets calibration diagrams respectively, which showed good consistency.

**Figure 5:**
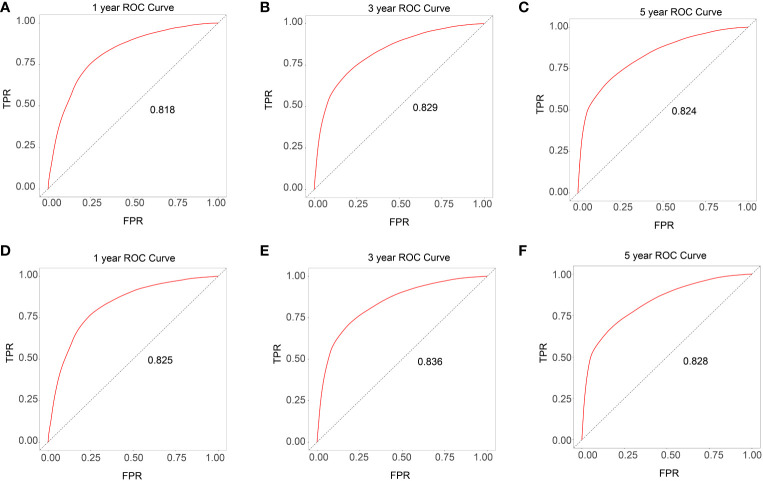
ROC curves for the training and validation data set (**A-C** training data set and **D-F** validation data set).

Based on the univariate and multivariate Cox regression analyses, a nomogram was constructed including all predictors (age, gender, race, site_recode_ICD, grade, CS_tumor_size, CS_extension, and metastasis) (**Figure 4A**). The calibration plot showed good agreement both in the training and validation datasets (**Figures 4B–F**). The AUC values of the 1-, 3-, and 5-year survival in the nomograms were 0.818, 0.829, and 0.824, respectively, in the training group (**Figures 5A–C**), while these values were 0.825, 0.836, and 0.828, respectively, in the validation group (**Figures 5D–F**). Furthermore, we calculated the C-index to assess the performance of the constructed nomograms. The predicted C-index values for the DSS nomogram were 0.787 and 0.782 in the training and validation datasets, respectively.

### 3.5 Clinical applicability of the nomogram

**Figure 6:**
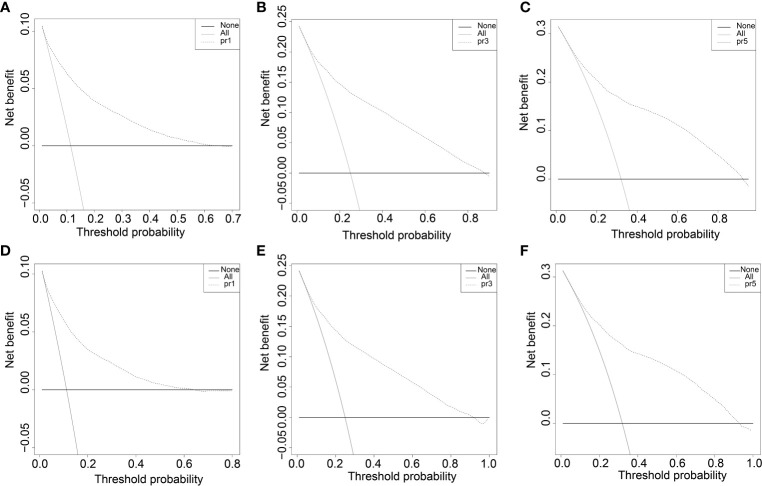
The decision curve (DCA) of DSS in training and validation data set. **(A-C)** DSS decision curve (DCA) of the training data set. D-F DSS Nomogram decision curve (DCA) of the validation data set.

The survival curves of the DSS of the 31,479 patients were plotted using the Kaplan–Meier method (**Figure 2A**). Our results illustrated that survival significantly decreased in COAD patients with follow-up time (*p* < 0.001). The DCA plots showed that the threshold probability was within the range from 0.1 to 0.9 with the maximum benefit range of the model (**Figures 6A–C**), which presented the same trend as the validation data with the Kaplan–Meier survival curves (*p* < 0.001) (**Figures 6D–F**) and consistent with the results for OS (**Supplementary Figure S6**).

## 4 Discussion

The nomogram was made simpler with multivariate regression analysis including many prognostic factors into a simplified estimation model constructed to predict the possibility of events (19, 20). The nomogram allows clinicians to more visually evaluate the individual health of patients and to offer personalized treatments (21, 22). At present, nomograms are commonly applied for prognosis (e.g., OS and DSS of patients with cancer) (23–25). A study found that *HOXC8*, *IRF7*, and *CXCL13* could be used as potential prognostic signatures for COAD based on the nomogram algorithm (26). Based on patients with COAD, we constructed a new prognosis prediction model.

The correlations between the clinical indicators were calculated. Survival_months and status had the most significant correlation for DSS (**Figure 1**), while metastasis and Survival_months showed the most significant correlation for OS (**Supplementary Figure S1**). This showed that metastasis was associated with prognosis. The independent prognostic factors for DSS and OS were confirmed *via* univariate and multivariate Cox regression analyses. Univariate analysis showed that age, gender, race, site_recode_ICD, grade, CS_tumor_size, CS_extension, and metastasis were associated with DSS (**Table 2**). These factors were then applied in the multivariate Cox regression. The results of the multivariate analysis showed that age, gender, race, site_recode_ICD, grade, CS_tumor_size, CS_extension, and metastasis were independent prognostic factors for both DSS and OS (**Table 3**, **Supplementary Table S1**). The HRs of gender, site_recode_ICD, grade, CS_tumor_size, and metastasis were higher than 1 for both DSS and OS (**Table 3**, **Supplementary Table S1**). This clarified that gender, site_recode_ICD, grade, CS_tumor_size, and metastasis were risk factors for COAD.

Kaplan–Meier survival analysis revealed that black race was significantly associated with the shortest DSS compared to other races (**Figure 2D**), implying that black patients need priority monitoring. The large intestine was significantly associated with the shortest DSS compared to others in site_recode_ICD (**Figure 2E**), indicating that more attention should be paid to this site. Early stage (stages I and II) was significantly associated with a higher DSS in site_recode_ICD (**Figure 2F**), and the risk increases with grade, which was in line with reality. CS_tumor_size was significantly associated with DSS (**Figure 2G**), and M1 metastasis showed a greater risk compared to M0 metastasis. In conclusion, the bigger the tumor_size and the more occurrence of tumor metastasis, clinical measures should be taken. The same results for DSS were found in the validation cohort (**Supplementary Figure S2**). Similarly, the same trends of the prognostic factors (age, gender, race, site_recode_ICD, grade, CS_tumor_size, CS_extension and metastasis) were also found for OS (**Supplementary Figure S3**, **S4**).

All independent prognostic factors in the Cox regression model analysis were used to build the prognostic prediction nomogram. By summing up the scores associated with each indicator variable according to the bottom scale by projecting the total points downward, the probabilities of OS and DSS at 1, 3, and 5 years were estimated for each patient. The C-index values indicated that our newly built nomogram had great potential to accurately predict the prognosis of patients. The DCA plots demonstrated good clinical utility in the training dataset for prediction of the 1-, 3-, and 5-year survival (**Figures 6A–C**). The validation set also showed similar trends (**Figures 6D–F**) and were consistent with the results of OS (**Supplementary Figure S6**). The DCA results revealed good predictive accuracy and clinical utility. However, the following study limitations remain. Firstly, this study had a retrospective design; therefore, the retrospective nature of this study cannot exclude all potential bias. Secondly, although we randomly split data into the training and validation datasets, more external validation, such as validation of the model in other institutions or other countries, is still necessary in the future.

In conclusion, we constructed and validated a nomogram model for predicting individualized survival probability in patients with COAD. This convenient visual nomogram showed not only excellent clinical utility but also the ability to adequately differentiate patients with COAD, suggesting that it may be a potentially simple and maneuverable tool for clinicians to personalize prognostic assessment and determine treatment strategies.

## Data availability statement

The original contributions presented in the study are included in the article/**Supplementary Material**. Further inquiries can be directed to the corresponding author.

## Ethics statement

This study is based on the SEER database and does not require ethical approval.

## Author contributions 

CY and WW performed the study and analyzed the data. CY wrote the manuscript. KH provided expert consultations and clinical suggestions. WC conceived the study. YC participated in the study design and coordination. XS helped draft the manuscript. All authors contributed to the article and approved the submitted version.

## References

[1] Siegel R Miller K Jemal A. Cancer statistics, 2020. CA: Cancer J Clin (2020) 70(1):7–30. doi: 10.3322/caac.21590 31912902

[2] Bray F Ferlay J Soerjomataram I Siegel RL Torre LA Jemal A. Global cancer statistics 2018: GLOBOCAN estimates of incidence and mortality worldwide for 36 cancers in 185 countries. CA Cancer J Clin (2018) 68(6):394–424. doi: 10.3322/caac.21492 30207593

[3] Wei W Zeng H Zheng R Zhang S An L Chen R. Cancer registration in China and its role in cancer prevention and control. Lancet Oncol (2020) 21(7):e342–e9. doi: 10.1016/S1470-2045(20)30073-5 32615118

[4] Feng RM Zong Y Cao S Xu R. Current cancer situation in China: Good or bad news from the 2018 global cancer statistics? Cancer Commun (London England) (2019) 39(1):22. doi: 10.1186/s40880-019-0368-6 PMC6487510 31030667

[5] Zheng RS Sun KX Zhang SW Zeng HM Zou XN Chen R. [Report of cancer epidemiology in China, 2015]. Zhonghua zhong liu za zhi [Chinese J oncol] (2019) 41(1):19–28. doi: 10.3760/cma.j.issn.0253-3766 30678413

[6] Siegel RL Miller KD Goding Sauer A Fedewa SA Butterly LF Anderson JC. Colorectal cancer statistics, 2020. CA Cancer J Clin (2020) 70(3):145–64. doi: 10.3322/caac.21601 32133645

[7] Doll K M RADEMAKER A SOSA JA. Practical guide to surgical data sets: Surveillance, epidemiology, and end results (SEER) database. JAMA Surg (2018) 153(6):588–9. doi: 10.1001/jamasurg.2018.0501 29617544

[8] Bonnett LJ Snell KIE Collins GS Riley RD. Guide to presenting clinical prediction models for use in clinical settings. Bmj (2019) 365(l737). doi: 10.1136/bmj.l737 30995987

[9] Li Y Liu W Zhou Z Ge H Zhao L Liu H. Development and validation of prognostic nomograms for early-onset locally advanced colon cancer. Aging (Albany NY) (2020) 13(1):477–92. doi: 10.18632/aging.202157 PMC7834989 33289705

[10] Zhang JX Song W Chen ZH Wei JH Liao YJ Lei J. Prognostic and predictive value of a microRNA signature in stage II colon cancer: A microRNA expression analysis. Lancet Oncol (2013) 14(13):1295–306. doi: 10.1016/S1470-2045(13)70491-1 24239208

[11] Balachandran VP Gonen M Smith JJ DeMatteo RP. Nomograms in oncology: More than meets the eye. Lancet Oncol (2015) 16(4):e173–80. doi: 10.1016/S1470-2045(14)71116-7 PMC4465353 25846097

[12] Jin Y Lin M Luo Z Hu X Zhang J. Development and validation of a nomogram for predicting overall survival of patients with cancer of unknown primary: A real-world data analysis. Ann Trans Med (2021) 9(3):198. doi: 10.21037/atm-20-4826 PMC7940932 33708825

[13] Hayat MJ Howlader N Reichman ME Edwards BK. Cancer statistics, trends, and multiple primary cancer analyses from the surveillance, epidemiology, and end results (SEER) program. Oncologist (2007) 12(1):20–37. doi: 10.1634/theoncologist.12-1-20 17227898

[14] Zhou ZR Wang WW Li Y Jin KR Wang XY Wang ZW. In-depth mining of clinical data: the construction of clinical prediction model with r. Ann Transl Med (2019) 7(23):796. doi: 10.21037/atm.2019.08.63 32042812 PMC6989986

[15] Collins GS Reitsma JB Altman DG Moons KG. Transparent reporting of a multivariable prediction model for individual prognosis or diagnosis (TRIPOD): The TRIPOD statement. Ann Intern Med (2015) 162(1):55–63. doi: 10.7326/M14-0697 25560714

[16] Moons KG Altman DG Reitsma JB Ioannidis JP Macaskill P Steyerberg EW. Transparent reporting of a multivariable prediction model for individual prognosis or diagnosis (TRIPOD): Explanation and elaboration. Ann Intern Med (2015) 162(1):W1–73. doi: 10.7326/M14-0698 25560730

[17] Fitzgerald M Saville B Lewis R. Decision curve analysis. Jama (2015) 313(4):409–10. doi: 10.1001/jama.2015.37 25626037

[18] Van Calster B Wynants L Verbeek JFM Verbakel JY Christodoulou E Vickers AJ. Reporting and interpreting decision curve analysis: A guide for investigators. Eur Urol (2018) 74(6):796–804. doi: 10.1016/j.eururo.2018.08.038 30241973 PMC6261531

[19] Lv Y Wang Z Li K Wang Q Bai W Yuan X. Risk stratification based on chronic liver failure consortium acute decompensation score in patients with child-pugh b cirrhosis and acute variceal bleeding. Hepatology (2020) 73(4):1478–93. doi: 10.1002/hep.31478 32706906

[20] Wu J Zhang H Li L Hu M Chen L Xu B. A nomogram for predicting overall survival in patients with low-grade endometrial stromal sarcoma: A population-based analysis. Cancer Commun (Lond) (2020) 40(7):301–12. doi: 10.1002/cac2.12067 PMC7365459 32558385

[21] Chen Q Mao R Zhao J Bi X Li Z Huang Z. Upgraded nomograms for the prediction of complications and survival in patients with colorectal liver metastases treated with neoadjuvant chemotherapy followed by hepatic resection. Ann Trans Med (2021) 9(3):265. doi: 10.21037/atm-20-3973 PMC7940886 33708892

[22] Lin Z Wang H Zhang Y Li G Pi G Yu X. Development and validation of a prognostic nomogram to guide decision-making for high-grade digestive neuroendocrine neoplasms. Oncologist (2020) 25(4):e659–e67. doi: 10.1634/theoncologist.2019-0566 PMC7160419 32297441

[23] Mao M Zhang A He Y Zhang L Liu W Song Y. Development and validation of a novel nomogram to predict overall survival in gastric cancer with lymph node metastasis. Int J Biol Sci (2020) 16(7):1230–7. doi: 10.7150/ijbs.39161 PMC7053322 32174797

[24] Jiang Y Li T Liang X Hu Y Huang L Liao Z. Association of adjuvant chemotherapy with survival in patients with stage II or III gastric cancer. JAMA Surg (2017) 152(7):e171087. doi: 10.1001/jamasurg.2017.1087 28538950 PMC5831463

[25] Lu YJ Duan WM. Establishment and validation of a novel predictive model to quantify the risk of bone metastasis in patients with prostate cancer. Transl Androl Urol (2021) 10(1):310–25. doi: 10.21037/tau-20-1133 PMC7844484 33532320

[26] Liao L Gao Y Su J Feng Y. By characterizing metabolic and immune microenvironment reveal potential prognostic markers in the development of colorectal cancer. Front Bioeng Biotechnol (2022) 10:822835. doi: 10.3389/fbioe.2022.822835 35992347 PMC9390973

