# Supplementary figures and images for: A novel prognostic model for patients with colon adenocarcinoma
Source: Front Endocrinol (Lausanne). 2023 Feb 27;14:1133554. doi: 10.3389/fendo.2023.1133554 (PMC10009111; doi:10.3389/fendo.2023.1133554)

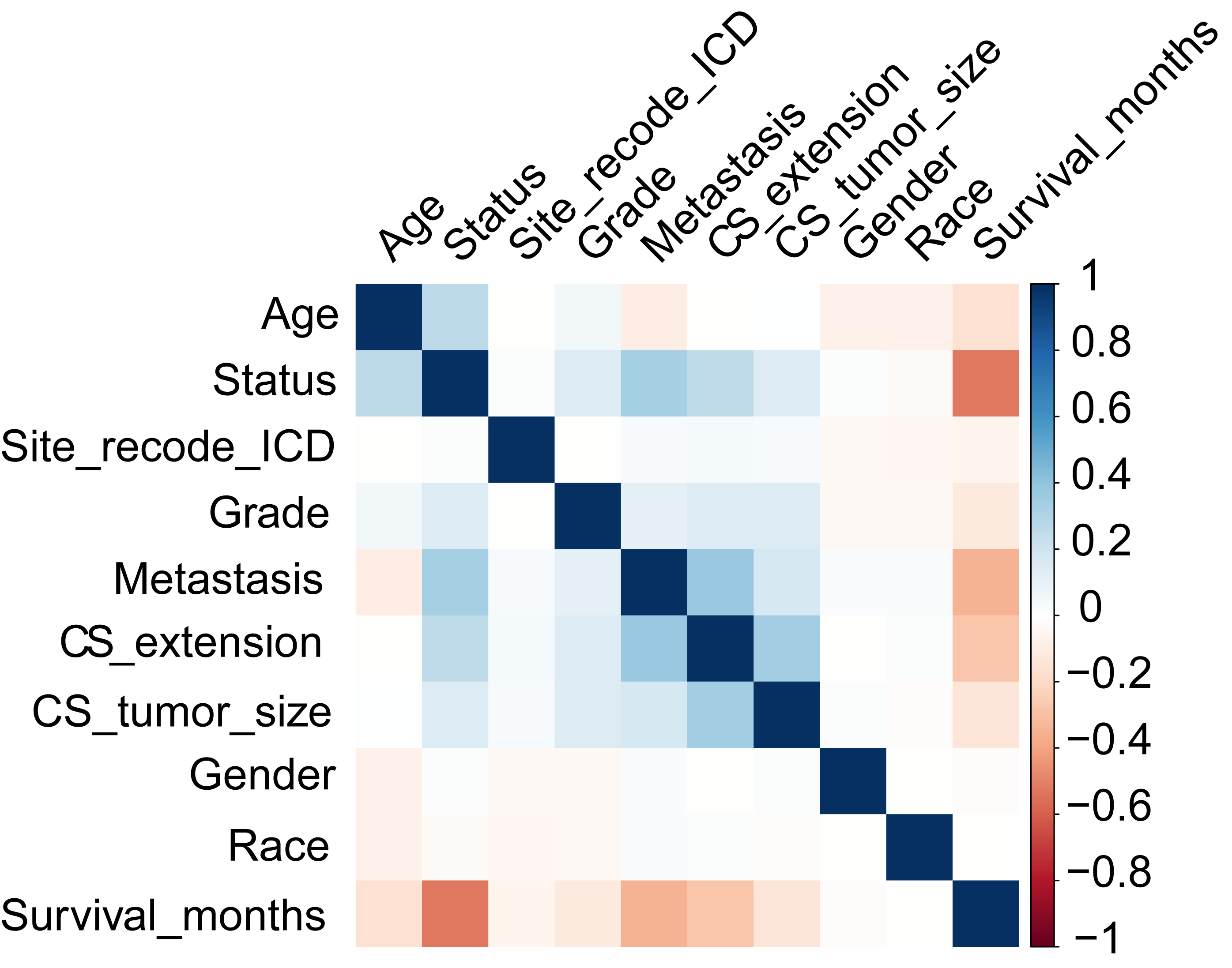

Supplement: Supplementary file 1 [file Image_1.tif]

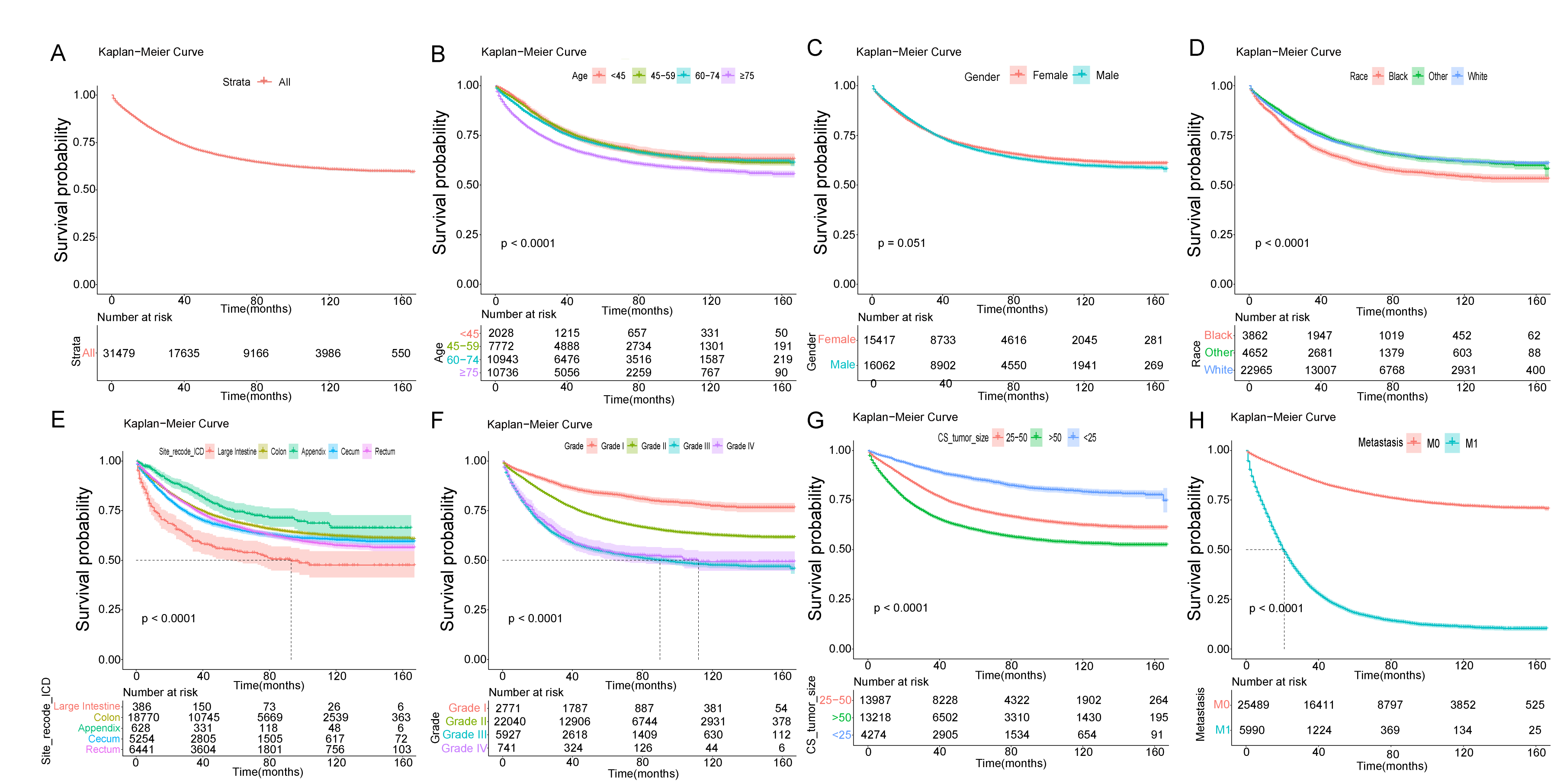

Supplement: Supplementary file 2 [file Image_2.tif]

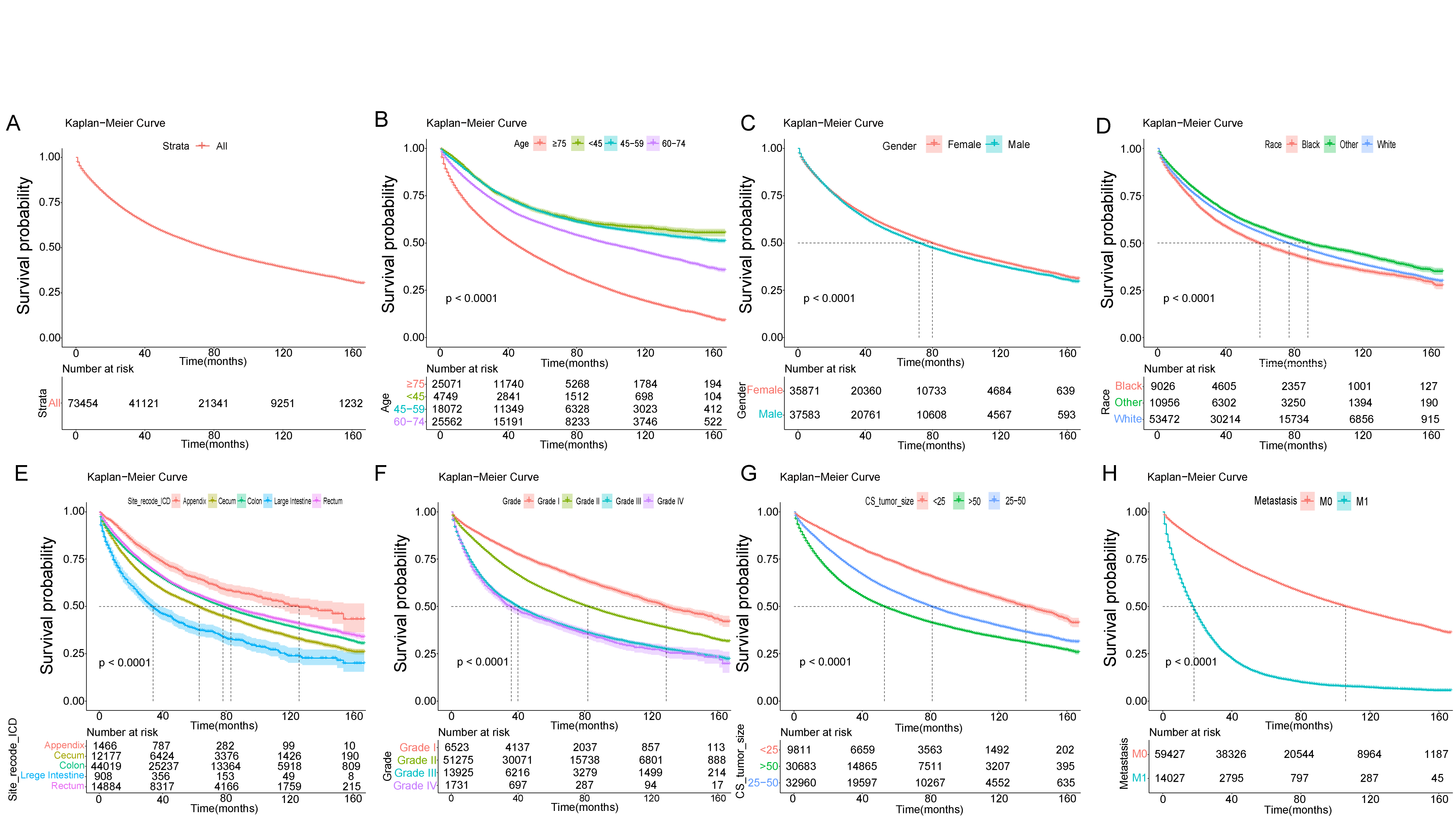

Supplement: Supplementary file 3 [file Image_3.tif]

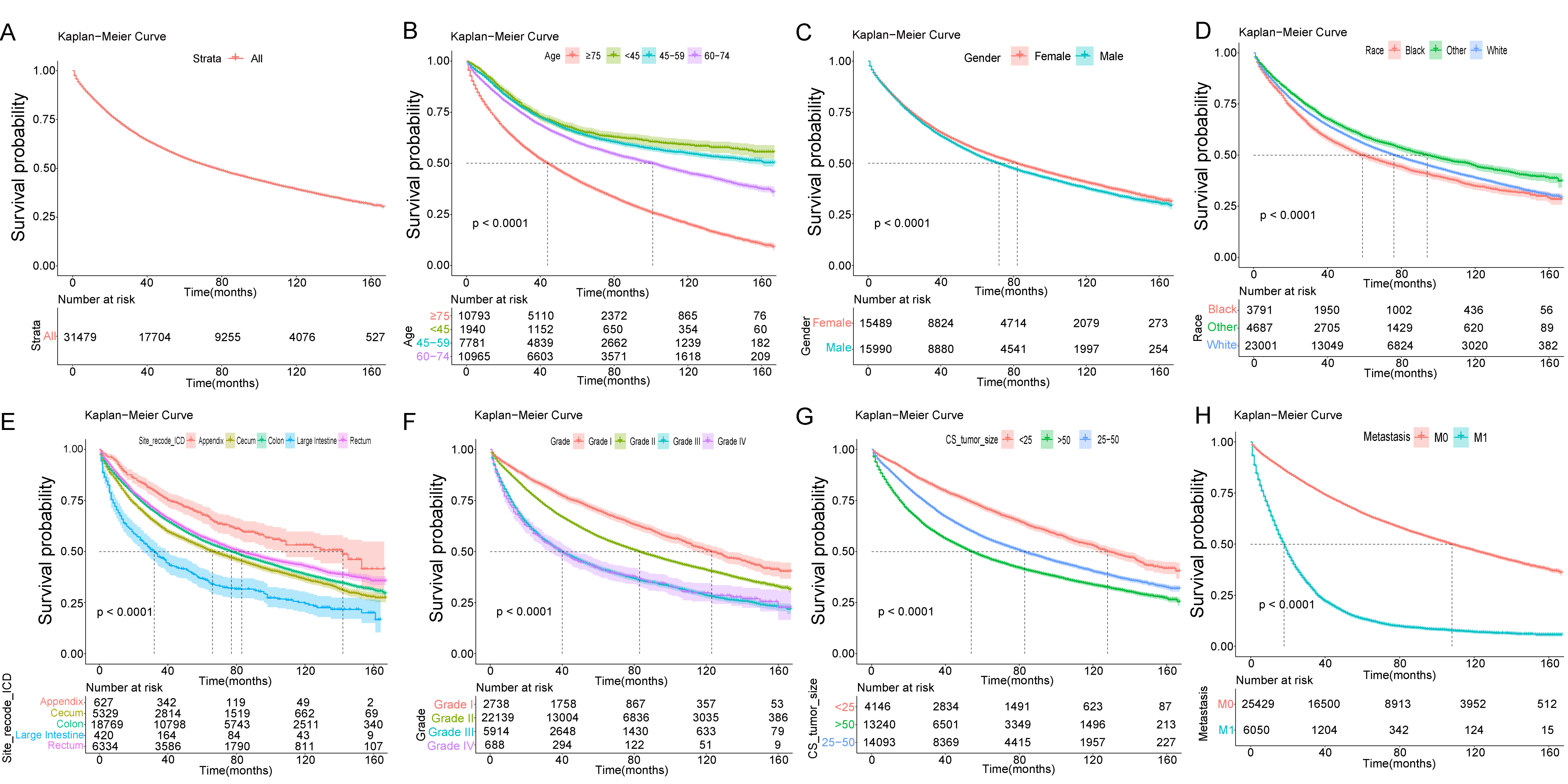

Supplement: Supplementary file 4 [file Image_4.tif]

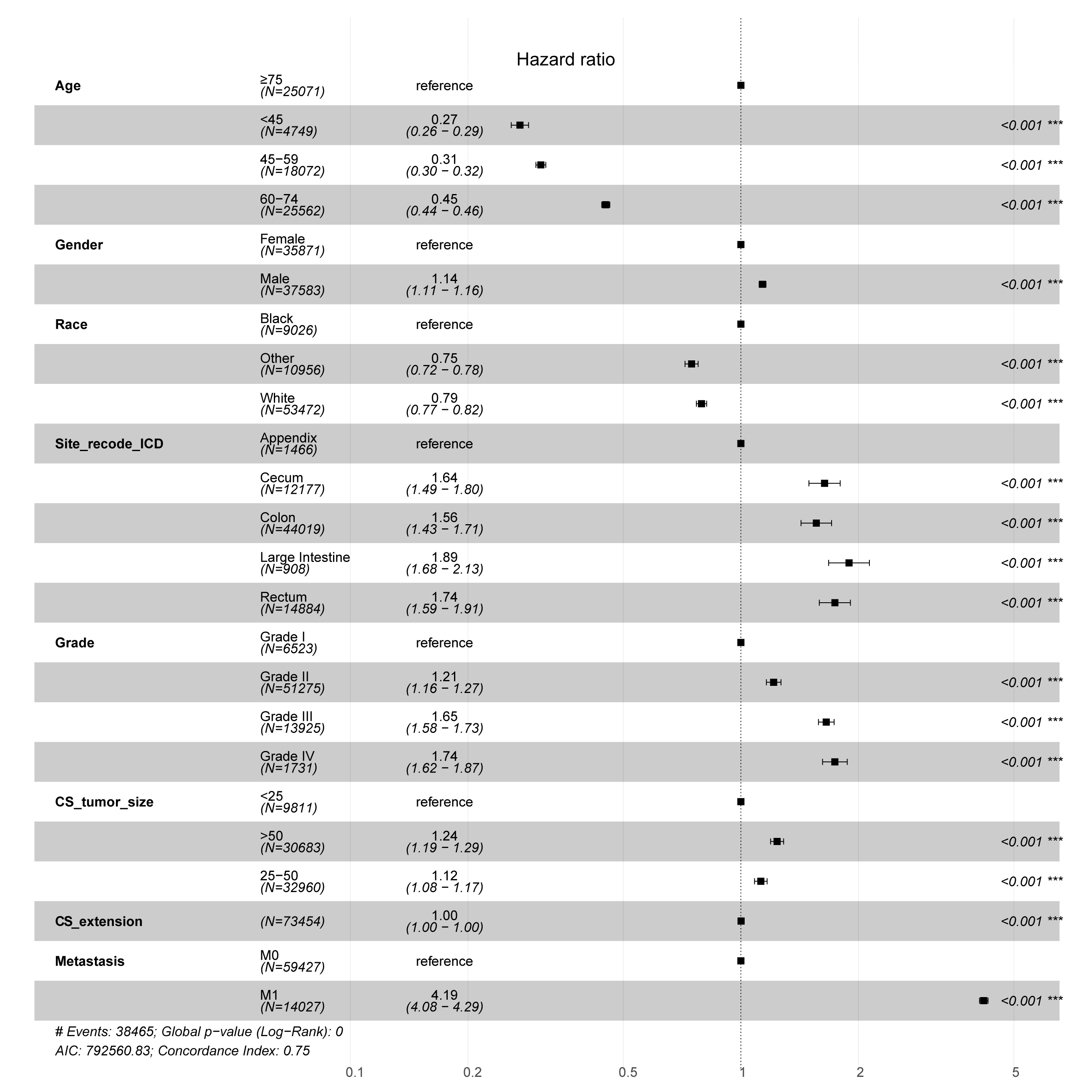

Supplement: Supplementary file 5 [file Image_5.tif]
