# Supplementary material for: A novel prognostic model for patients with colon adenocarcinoma
Source: Front Endocrinol (Lausanne). 2023 Feb 27;14:1133554. doi: 10.3389/fendo.2023.1133554 (PMC10009111; doi:10.3389/fendo.2023.1133554)
Supplement: Supplementary file 7 [file Table_1.docx]

**Table S1 OS Multivariable cox analysis**

| **Variable** | **HR** | **95% CI** | | **P value** |
| --- | --- | --- | --- | --- |
|  |  | **Lower** | **Upper** |  |
| Age(years) |  |  |  |  |
| ≥75 | Reference |  |  |  |
| <45 | 0.272 | 0.258 | 0.286 | <0.001 |
| 45-59 | 0.308 | 0.299 | 0.317 | <0.001 |
| 60-74 | 0.451 | 0.440 | 0.462 | <0.001 |
| Gender |  |  |  |  |
| Female | Reference |  |  |  |
| Male | 1.136 | 1.113 | 1.159 | <0.001 |
| Race |  |  |  |  |
| Black | Reference |  |  |  |
| White | 0.793 | 0.769 | 0.817 | <0.001 |
| Other | 0.748 | 0.719 | 0.777 | <0.001 |
| Site_recode_ICD |  |  |  |  |
| Appendix | Reference |  |  |  |
| Cecum | 1.637 | 1.493 | 1.796 | <0.001 |
| Colon | 1.560 | 1.426 | 1.707 | <0.001 |
| Large Intestine | 1.892 | 1.678 | 2.133 | <0.001 |
| Rectum | 1.741 | 1.588 | 1.908 | <0.001 |
| Grade |  |  |  |  |
| Grade I | Reference |  |  |  |
| Grade II | 1.213 | 1.162 | 1.267 | <0.001 |
| Grade III | 1.654 | 1.580 | 1.733 | <0.001 |
| Grade IV | 1.741 | 1.619 | 1.872 | <0.001 |
| CS_tumor_size |  |  |  |  |
| <25 | Reference |  |  |  |
| >50 | 1.238 | 1.192 | 1.286 | <0.001 |
| 25-50 | 1.125 | 1.084 | 1.167 | <0.001 |
| CS_extension | 1.001 | 1.001 | 1.002 | <0.001 |
| Metastasis |  |  |  |  |
| M0 | Reference |  |  |  |
| M1 | 4.187 | 4.083 | 4.293 | <0.001 |

HR, hazard ratio; CI, confidence interval

“Other” in the Race : American Indian (AK Native) and Asian (Pacific Islander).
